# Creatinine and cystatin C: Shooting at a flying target

**DOI:** 10.3109/03009734.2011.577918

**Published:** 2011-06-29

**Authors:** Chia-Ter Chao

**Affiliations:** Division of Nephrology, Department of Internal Medicine, National Taiwan University Hospital, College of Medicine, National Taiwan University, Taipei, Taiwan

Dear Sir,

I read with great interest the article entitled ‘Significant differences when using creatinine, modification of diet in renal disease, or cystatin C for estimating glomerular filtration rate in ICU patients’ by Lipcsey et al. (1) in the March 2011 issue of the *Upsala Journal of Medical Sciences*. This article provides an interesting analysis of estimating renal function in critically ill patients, but I believe there are several aspects that may warrant further discussion:

First of all, the equations estimating glomerular filtration rate (GFR) are all notoriously flawed in the setting of rapidly changing renal function, that is, acute kidney injury. The creatinine-based formula for creatinine clearance is shown to over-estimate renal function when actual GFR is low, due to its multiple interfering factors and tubular secretion nature, but the cystatin C-based formula is also affected by muscle mass and adiposity (2). To complicate things further, serum cystatin C level is confounded by inflammation status and corticosteroid use, both of which are frequently found in patients under critical care (3). With this in mind, we can presume that both formulae will not suffice as good estimators of renal function in critically ill patients with acute kidney injury, especially when patients are admitted with relative adrenal insufficiency or septic shock status.

Second, the duration of illnesses and timing of tests are also not taken into consideration in Lipcsey's article. Nejat et al. in their multicenter observation study have indicated that cystatin C rises earlier than creatinine in patients with acute kidney injury by 4–5 hours from a general ICU population (4). Herget-Rosenthal et al. also demonstrated a lag time, on average one-and-a-half days, between cystatin C elevation and creatinine to the same level (5). In this sense, the significant difference between cystatin C and creatinine-estimated GFR in Lipcsey's results will be prominently influenced by the timing of blood tests. If intensivists elect to check renal markers earlier in the course of disease by hours or days, they will undoubtedly discover a sharp difference between actual and estimated renal function, while later tests will not. This is also reflected in the study by Endre et al., in which a biomarker for acute kidney injury achieves better predictability of outcome when it is tested earlier (6).

Finally, is there any better way of determining renal function in the critically ill with convenient point-of-care feasibility? Tidman et al. in their study of GFR determination in chronic kidney disease patients have provided a new thought: combining creatinine-based and cystatin C-based results for estimation (7). Though this study focuses on patients with stable renal function, it may be worthwhile to extrapolate this finding to the critically ill ones. To target flying objects, we need to trace more accurately their path and take it with one shot.

## References

[1] Lipcsey M, Furebring M, Rubertsson S, Larsson A (2011). Significant differences when using creatinine, modification of diet in renal disease, or cystatin C for estimating glomerular filtration rate in ICU patients. Ups J Med Sci.

[2] Bagshaw SM, Bellomo R (2010). Cystatin C in acute kidney injury. Curr Opin Crit Care.

[3] Rule AD, Bergstralh EJ, Slezak JM, Bergert J, Larson TS (2006). Glomerular filtration rate estimated by cystatin C among different clinical presentations. Kidney Int.

[4] Nejat M, Pickering JW, Walker RJ, Endre ZH (2010). Rapid detection of acute kidney injury by plasma cystatin C in the intensive care unit. Nephrol Dial Transpl.

[5] Herget-Rosenthal S, Marggraf G, Hüsing J, Göring F, Pietruck F, Janssen O (2004). Early detection of acute renal failure by serum cystatin C. Kidney Int.

[6] Endre ZH, Pickering JW, Walker RJ, Devarajan P, Edelstein CL, Bonventre JV (2011). Improved performance of urinary biomarkers of acute kidney injury in the critically ill by stratification for injury duration and baseline renal function. Kidney Int.

[7] Tidman M, Sjöström P, Jones I (2008). A comparison of GFR estimating formulae based upon s-cystatin C and s-creatinine and a combination of the two. Nephrol Dial Transpl.

